# The life of the cortical column: opening the domain of functional architecture of the cortex (1955–1981)

**DOI:** 10.1007/s40656-016-0103-4

**Published:** 2016-06-17

**Authors:** Philipp Haueis

**Affiliations:** 1 Max Planck Research Group for Neuroanatomy & Connectivity, Max Planck Institute for Human Cognitive and Brain Sciences, Stephanstr. 1a, 04103 Leipzig, Germany; 2 Berlin School of Mind and Brain, Luisenstr. 56, 10117 Berlin, Germany

**Keywords:** Cortical column, Conceptual development, Experiment, History of neuroscience, Mountcastle, Hubel and Wiesel

## Abstract

The concept of the cortical column refers to vertical cell bands with similar response properties, which were initially observed by Vernon Mountcastle’s mapping of single cell recordings in the cat somatic cortex. It has subsequently guided over 50 years of neuroscientific research, in which fundamental questions about the modularity of the cortex and basic principles of sensory information processing were empirically investigated. Nevertheless, the status of the column remains controversial today, as skeptical commentators proclaim that the vertical cell bands are a functionally insignificant by-product of ontogenetic development. This paper inquires how the column came to be viewed as an elementary unit of the cortex from Mountcastle’s discovery in 1955 until David Hubel and Torsten Wiesel’s reception of the Nobel Prize in 1981. I first argue that Mountcastle’s vertical electrode recordings served as criteria for applying the column concept to electrophysiological data. In contrast to previous authors, I claim that this move from electrophysiological data to the phenomenon of columnar responses was concept-laden, but not theory-laden. In the second part of the paper, I argue that Mountcastle’s criteria provided Hubel Wiesel with a conceptual outlook, i.e. it allowed them to anticipate columnar patterns in the cat and macaque visual cortex. I argue that in the late 1970s, this outlook only briefly took a form that one could call a ‘theory’ of the cerebral cortex, before new experimental techniques started to diversify column research. I end by showing how this account of early column research fits into a larger project that follows the conceptual development of the column into the present.

## Introduction

 The cortical column designates vertical structures that span all layers of the cerebral cortex, and in which neurons show similar responses to sensory stimuli. The original definition of the column goes back to Vernon Mountcastle’s mapping of single-cell recordings in the somatic sensory cortex of the cat (Mountcastle et al. 1955). It has subsequently become a basic concept that has guided over 50 years of neuroscientific research (Shepherd 2010), in which fundamental questions about the modularity of the cortex and basic principles of sensory information processing were empirically investigated. After physiologists successfully applied the column concept to many sensory and some “higher” areas of brain function, it came to be viewed as an “elementary unit of organization” of the entire cerebral cortex (Mountcastle 1957, p. 430; see also 1978, p. 16; 1997, p. 701).

Compared to its older conceptual relatives such as “neuron” (Waldeyer-Hertz 1891) or “cortical area” (Brodmann 1909), however, the status of the column as a basic organizational unit of the cortex remains controversial.^1^ Commemorating the 50th anniversary of Mountcastle’s discovery, the visual neuroscientists Jonathan Horton and Daniel Adams published a review in which they came to “the disappointing realization that the column may have no function” (Horton and Adams 2005, p. 837). Their claim rested on conceptual ambiguities, conflicting evidence and cross-species comparisons that seemed irreconcilable with viewing the column as a basic unit of the cortex. One prominent neuroanatomist subsequently noted that there is no agreed-upon, general definition of cortical columns (Rakic 2008). In turn, several authors proposed to redefine the column concept (Rockland 2010) or replace it altogether (da Costa and Martin 2010). The status of the column concept therefore remains an open issue in current neuroscientific practice.

From the perspective of history and philosophy of science, the case of the column poses—at least—the following three questions: How did the column come to be regarded a basic concept of neuroscientific research? Which subsequent experimental results conflicted with its status as an organizational unit of the cortex and why? And given its uncertain status today, what is at stake in currently using the column concept to investigate cortical organization? The following paper attempts to answer the first of these questions as a step towards a larger project that follows the conceptual development of the column into the present. It thereby combines the approach of historians of science to follow the historical trajectories of scientific entities (Rheinberger 1997; Daston 1999) with the aim of philosophers of neuroscience to “examine the issues raised by central concepts of neuroscience” (Chirimuuta and Gold 2009, p. 200). In the metaphorical diction of the title, this paper describes the “birth” of the column in neuroscientific practice.^2^

In the first part of the paper, I show how Mountcastle established experimental conditions that allowed him to move from single cell recordings to the *phenomenon* of a columnar response pattern in neuronal populations. In contrast to Hardcastle and Stewart (2003), who describe this move in electrophysiology as “theory-laden,” I suggest that in the case of the column, this process is better captured by the term “concept-laden.” In the second part of the paper, I argue that Mountcastle’s criteria provided David Hubel and Torsten Wiesel with a *conceptual outlook* (Lange 2000), i.e. it allowed them to anticipate columnar patterns in the cat and macaque visual cortex. I argue that in the late 1970 s, this outlook only briefly took a form that one could call a “theory” of the cerebral cortex, before new experimental techniques started to diversify column research. In support of the latter claim, I end by showing how the columnar outlook started to get revised in response to new experimental results, shortly before Hubel and Wiesel received the Nobel prize in 1981. I conclude by sketching how the early history of column research fits into a bigger narrative about the subsequent “maturity” and the proclaimed “death” of the column by Horton and Adams in 2005. This approach to focus on concept and concept-ladenness of observation emphasizes the patchy and sometimes unpredictable trajectories of how concepts are applied in changing empirical circumstances (see also Wilson 2006; Rouse 2015). Investigating the role of concepts in ongoing research can therefore help philosophers and historians to detect fine-grained changes of conceptual application that may be easily overlooked by an exclusive focus on general theories or textbook treatments of central neuroscientific concepts.

## Mountcastle’s discovery of the column

### Opening a domain of inquiry: Mountcastle’s early research on the column (1955–1959)

According to Mountcastle’s own recollections, the history of the column started on a “yellow piece of paper.” Noting down results from electrode recordings in vertical lists it seemed like cells that responded to similar external stimuli of the sensory periphery formed vertical rows through the cortical layers (Grauer 2007). After inserting an electrode perpendicular to the cortical surface, Mountcastle’s group recorded responses of individual cells from the region of the cat somatic cortex, where afferent fibers of the cat’s skin receptors terminated. Strikingly, most units encountered in individual penetrations responded to the same spot and kind of skin stimulation throughout the entire gray matter. The researchers therefore tentatively concluded that their results indicated “a vertical organization of columns of cortical cells for topography and modality” (Mountcastle et al. 1955, p. 647).

A technical challenge for such in vivo recordings was that cardiac and respiratory activity moved the brain surface, so that cell positions shifted relative to the fixed microelectrode. Philipp Davies’ construction of a liquid-filled recording chamber solved this problem because the liquid absorbed the movement by covering the skull-free cortex (Davies 1956). Davies acquired the technical know-how for building this chamber from his previous work with biophysicist Detlev Bronk, in which they studied changes in oxygen concentrations at the cortical surface. The cardiac effects that produced such changes were also the source of the vascular pulsations that needed to be eliminated as noise in Mountcastle’s experimental system. In addition, Davies mounted the electrode to a microdrive on top of the chamber so that it could be lowered into any point of the cortical tissue below (Davies and Brink 1942). With this new technical apparatus, Mountcastle et al. (1957) went on to define stable recording conditions to apply the column concept in vertical penetrations. In the closed-chamber system, the length of the experiment was “usually under the control of the observer” (ibid., 389), and the researchers could record from single cells in the anesthetized cat for hours.^3^

Based on these recording conditions, Mountcastle (1957) now classified the measured neurons of the cat somatic cortex into functional types. He defined different “modalities” for units rising fast in spiking frequency and exhibiting a steady state of continuous spike trains when either the cat’s hair was depressed, its skin was pressured, the joints were rotated or deep fascia tissue was stimulated. Nearly perpendicular electrode penetrations recorded cells that all optimally responded to the same modality. The area of the stimulated body surface to which the cells responded also remained almost constant, whereas penetrations at a 45º angle shifted the peripheral fields gradually. Mountcastle therefore concluded that “neurons which lie in vertical columns extending across the layers belong usually to one and the same modality type” (ibid., 423). Based on the peripheral field shifts in the recordings at 45º angle, Mountcastle inferred that the cylindrically shaped columns must be maximally 0.5 mm wide. Because adjacent vertical penetrations also alternated in modality, he assumed that the columns were arranged in an intermingling mosaic, perhaps determined by the distribution of modality-specific fibers from the thalamus.

Mountcastle’s idea of a vertical column was met with so much skepticism that even his own co-workers requested not to appear as authors on the paper (cf. Mountcastle 2009, p. 359). At the time, the dominant neuroanatomical view focused on horizontal layers as revealed by cytoarchitectonics and its Nissl-stained cell bodies. It was thus even worse that Mountcastle and his colleagues could not find the electrode tracks in most post-mortem sections of the examined cat brains. Short of anatomical evidence, Mountcastle resorted to the work of Rafael Lorente de Nó (1949), who had inferred a vertical connectivity pattern of neurons from Golgi staining of the mouse somatosensory cortex.^4^ De Nó’s postulated anatomical unit was precisely what Mountcastle needed to count subsequent vertical cell recordings as elements of the same pattern of *columnar* activity. The final piece of indirect support came from vertical lesion studies in which cats did not show visual discrimination deficits (Sperry 1955), suggesting that higher-order functions could be implemented independently of intact horizontal connections.

To alleviate initial skepticism further, Mountcastle and neuroanatomist Tom Powell repeated the effort to reconstruct electrode tracks after recording vertically, this time through the postcentral gyrus of the macaque (i.e. its somatosensory area). Again, “the majority of the electrode tracks were extremely difficult to find” (Powell and Mountcastle 1959, p. 135). A magnified photograph of the exposed cortical surface was used to mark the insertion spot of the electrode, but the standard procedure of tissue slicing cut the electrode tracks into many small elements that became almost impossible to distinguish from small blood vessels or damaged glia cells. In order to avoid this histological jigsaw puzzle, Powell and Mountcastle inserted two thin wires along the micropipette. But even with these additional markers, the location of the electrode remained elusive. Powell had to use a vascular landmark on the photograph of the cortical surface so that in the first section, the electrode entry could be found among a vast network of branching arteries and fusing veins.

**Fig. 1:**
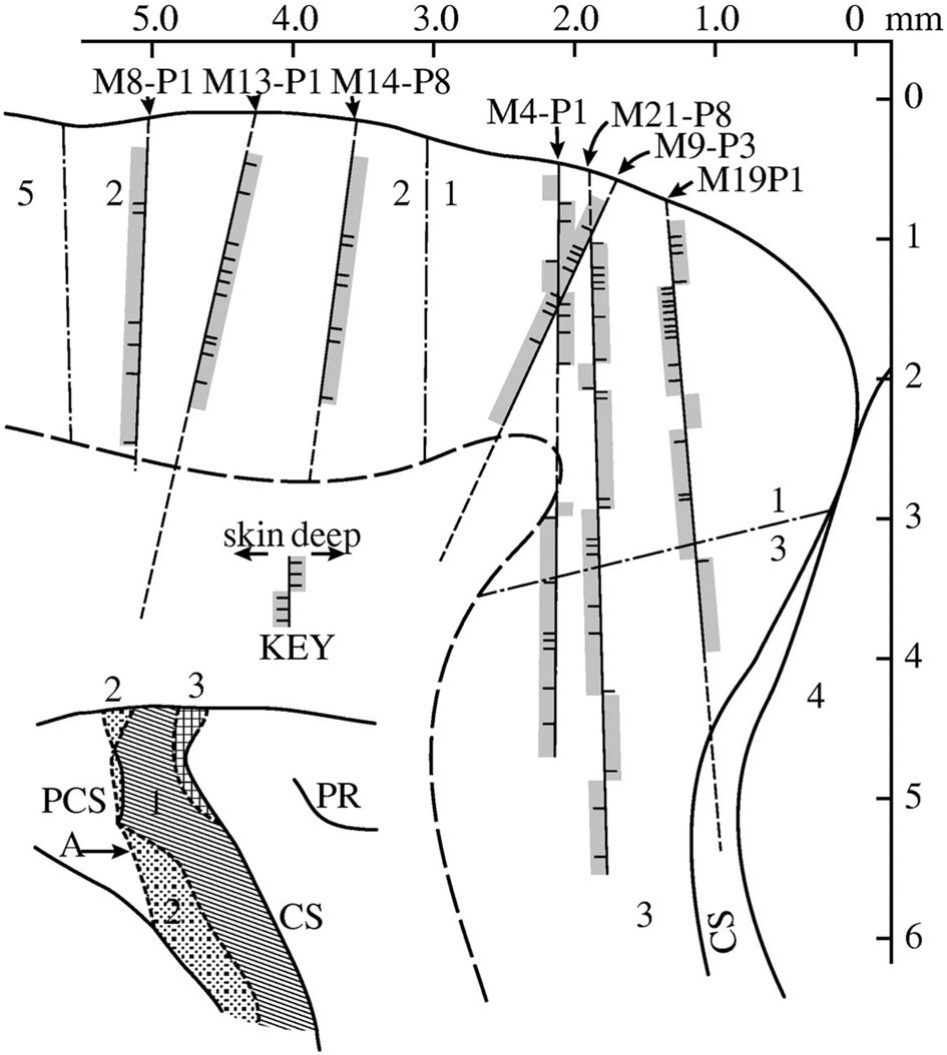
Track reconstructions of vertical electrode recordings in macaque areas *2* (*upper left*), *1* (*upper right*) and *3* (*lower right*) (Powell and Mountcastle 1959, Fig. 6). *M9-P3* shows how a perpendicular penetration produces modality-pure responses among mixed results of oblique penetrations. *M21-P8* and *M19-P1* are examples of opposing modality responses in parallel recordings through area 3 (between 3 and 4 mm depth)

The detailed report Powell and Mountcastle gave of reconstruction techniques shows the importance of *anatomical skills* to make proper inferences from electrophysiological recordings. The researchers now hypothesized that if the recording chamber was to be placed on the flat surface of macaque area 1 and 2, almost vertical recordings should result in modality-pure columnar response patterns. A 90º change in the vertical axis of area 3 should result in mixed modality responses. In agreement with the slowly bending surface, perpendicular penetrations showed most modality pure recordings in area 2, decreasing in area 1 before becoming highly intermixed in area 3. Given that the modality shifts in area 3 formed blocks of similar responses, the researchers also inferred that the electrode successively passed through parallel, modality-pure columns (cf. Powell and Mountcastle 1959, p. 147, 159; see Fig. 1 below). Nevertheless, the anatomical reconstructions of the electrode tracks did not show any anatomical borders which would delineate the modality changes occurring every 500 µm. Despite all technical and methodological improvements to control the responses of the experimental system, the column would still not show itself anatomically as a vertically connected cell band.

### From data to phenomena: experiments, technology and conceptual application

The philosophical question posed by these studies is how Mountcastle moved from observations of electrical cell responses to the claim that the cat somatosensory cortex exhibits a columnar organization. A promising starting point is to characterize this move in terms of Bogen and Woodward’s (1988) distinction between data and phenomena. Data are observable measurement outcomes that are produced in a local experimental context. An example of data from early column research are the optimal single unit responses measured with microelectrodes while stimulating the cat’s skin. The phenomenon, in contrast, is the vertical arrangement of somatosensory neurons of the same modality type that exhibits a columnar response pattern upon peripheral stimulation in different species.

Bogen and Woodward (1988, p. 306) hold that while experimental data are *observed*, phenomena like the columnar response pattern are *explained* by the theories about the investigated domain. In the experiments so far described, however, theoretical considerations were conspicuously absent, because the basic concepts for describing the explanandum phenomenon had not yet been articulated (cf. Rouse 2015, p. 223ff.; pp. 301–321). Mountcastle instead introduced the concept of the column to develop an understanding of an unknown research domain which Hubel and Wiesel later referred to as the *functional architecture of the cortex*. Well-developed theories also did not play a crucial role quite generally in the kind of electrophysiological research Mountcastle pursued. There was no theory that guided researchers to find out whether their recordings contained reliable data describing phenomena like neuronal spike trains, or the columnar response patterns of many such trains (cf. Hardcastle and Stewart 2003, p. 204). Therefore, researchers had to rely on previous observations and experience, rather than theory, to guide their analysis of newly recorded data. Mountcastle et al. (1957) did exactly that when they decided to exclude initially positive discharges as signs of cell damage (cf. ibid., 386). In previous studies, positive discharges always occurred when the electrode tip moved forwards, hinting at a contact with the cell membrane. Unlike Hardcastle and Stewart (2003, p. 303), however, I do not think that such “skillful guesswork” made the observation of electrophysiologists “theory-laden”. Against this broad conception of theory-ladenness, I emphasize that the success of finding columnar response patterns depended on technological conditions, like proper anesthetic dosages, movement reduction by the liquid in the chamber and reliable anatomical electrode tracks.^5^ The only experimental condition that matches Hardcastle and Stewart’s description is the *placement* of the recording chamber perpendicular to the cortical surface. But the condition is better described as *concept*-*laden*, because it was the column concept that implied that the recording angle had to be perpendicular in order to yield the requisite data.

Put differently, the experimental design of the early column studies supports the semantic thesis that experimental conditions offer *criteria* for detecting whether empirical concepts apply in scientific practice or not (see also Haueis 2014b). The recording chamber, and its orientation, is the technological enabling condition for recognizing columnar patterns in the negative discharges from single unit responses to peripheral stimulation. Histological traces of penetrations performed at a defined angle allowed for (limited) inferences about the anatomical arrangement of these cells. The material configuration of the conditions according to the expectation of a vertical response pattern makes the subsequent observations concept-laden, but not necessary theory-laden. Had the observations been theory-laden, one would expect the experimental conditions be set up to test particular (causal) hypotheses, which was not the case in Mountcastle’s early experiments.

But the crucial point about the data-phenomena distinction is perhaps not that phenomena are theory-laden, but rather underwritten by *mechanistic* explanations. To some extent, the column experiments look like an investigation of a multi-level mechanism (Craver 2007). Stimulus-specific neurons are organized spatially (i.e. vertically) and temporally (short latency times) such that as a whole, they exhibit a columnar response pattern. This description, however, does not properly reflect the state of column research circa 1959. The correlational techniques of single cell recording were not sufficient to establish an explanatory causal relation between stimulus, single unit recordings and the observed columnar patterns. Mountcastle’s own attempts to explain how cats can discriminate two points on the sensory surface, or how they sense their own position did not refer to columns at all (cf. Mountcastle 1957, p. 427, 429). The column concept could not be used in such explanations because its application criteria were restricted to laboratory conditions: “In the normal waking animal the mechanical deformations of peripheral tissues which occur are not so discretely arranged as they are by the experimenter” (ibid., p. 428).

In absence of both theory and mechanistic explanations, the early stage of column research is perhaps more adequately described as being concerned with the creation of a new phenomenon under laboratory conditions (Hacking 1983). The Mountcastle group introduced new instruments and measurements and accepted their readings as evidence for the existence and behavior of a new entity (cf. Haugeland 2013, p. 173), i.e. the cortical column exhibiting modality pure responses. The purpose of their experiments was to show that such responses were a detectable and hence intelligible phenomenon at all. Mountcastle’s research had therefore opened up a new domain of inquiry.

## Hubel and Wiesel’s columnar outlook

### Extending the domain: the study of columns in the visual cortex (1962–1974)

Hubel and Wiesel’s study of the column did not start with a list of notes on paper, as in Mountcatle’s case, but with a sound over a speaker. When they used Stephen Kuffler’s stimulus of circular light and dark spots while listening to cellular responses over an audiomonitor, the neurons in the cat’s visual cortex remained silent (these negative results where reported a year later in Hubel and Wiesel 1959, p. 587). After the researchers had again been recording from a single cellular unit for several hours in 1958, they slid a black dot into the ophthalmoscope “when suddenly over the audiomonitor the cell went off like a machine gun” (Hubel 1981, p. 27). Upon further inspection, they guessed that moving the glass edge of the slide created a thin shadow on the eye to which the cell responded optimally. The angle of movement could only be varied slightly before responses were lost. From that moment on, the concept of *orientation selectivity* would guide their subsequent research into the response properties of visual neurons.

To investigate orientation selectivity more systematically, Hubel and Wiesel could rely on Hubel’s earlier work with awake and unrestrained cats, which had shown that the organization of receptive fields remained unaffected by anesthesia.^6^ These results supported the experimental procedures in the visual cortex, which receives peripheral visual input from the same structure: the lateral geniculate nucleus (LGN). Mountcastle could infer that somatosensory neurons responded to either skin or deep modalities because they receive input from distinct peripheral receptors via different thalamic pathways. In contrast, Hubel and Wiesel had to infer the link of orientation-selective visual neurons to the LGN from differences in the functional responses alone. Therefore, their experimental success crucially depended on ruling out that functional responses in the cortex were artifacts induced by anesthesia. By comparing such responses to previous experiments on the functional properties of LGN cells, Hubel and Wiesel (1962, p. 123) could distinguish *simple* and *complex* cells in the cat visual cortex. Like LGN cells, simple cells had receptive fields with excitatory and inhibitory regions in which a light spot increases or decreases firing, respectively. The shape of the fields was elongated however, so that a narrow, orientation-specific bar would produce an optimal response (cf. ibid., 110). Because such cells most frequently occurred in layer 4, the researchers inferred that they received input from several LGN cells whose circular “on” centers were successively distributed throughout the rectangular field. Such a wiring scheme could also elucidate the complex cells like the 9 h “machine gun” neuron of 1958. Complex cells were orientation-specific but neither showed position-specific or antagonistic responses like simple cells. Hubel and Wiesel argued that they must receive feedforward excitations from simple cells with similar orientations but different visual field positions to show such responses (cf. ibid., p. 143).

In the recordings, simple and complex cells with common orientation axis tended to be grouped together when the electrode was inserted perpendicular to the cortical surface (cf. ibid., Figure 13, p. 131). Whereas most simple cells occurred in layers 3, 4 and 6, almost no complex cells were found in layer 4, suggesting that neurons that primarily receive lateral geniculate input had simple receptive field characteristics (cf. ibid. 138ff., 142). The recordings also suggested that orientation selective neurons were organized into columns, whose cortical surface width was estimated at 0.5 mm, although their shape appeared to be “very irregular” (cf. ibid, p. 131). Despite the uncertainty about column shape, the observed response patterns and the putative wiring scheme became mutually reinforcing:

We see at once that gathered together in discrete columns are the very cells we require to be interconnected in our scheme […] The otherwise puzzling aggregation of cells with common axis orientation now takes on new meaning. We may tentatively look upon each column as a functional unit of cortex, within which simple fields are elaborated and then in turn synthesized into complex fields (ibid., p. 144).

The distribution of simple and complex cells along a perpendicular recording gave Hubel and Wiesel’s wiring scheme an experimental manifestation: simple receptive fields in layer 4 were elaborated into more complex characteristics in the superficial and deep layers. But at the same time, the wiring scheme provided a justification to apply the column concept to the perpendicular recordings. Unlike in the somatosensory cortex, orientation selectivity was not linked to different peripheral receptors that segregated the columns. The wiring scheme specified the intracortical connections to make the segregation of orientation axes into columns anatomically plausible. The mutually reinforcing character of the recordings and the wiring scheme points to the importance of concepts in understanding the epistemology of experimental evidence. The concept-ladenness of observing functional response properties vertically proved to be advantageous: in the absence of anatomical connectivity data, Hubel and Wiesel could only show that their wiring scheme was plausible by locating it in functional electrode recordings.

**Fig. 2:**
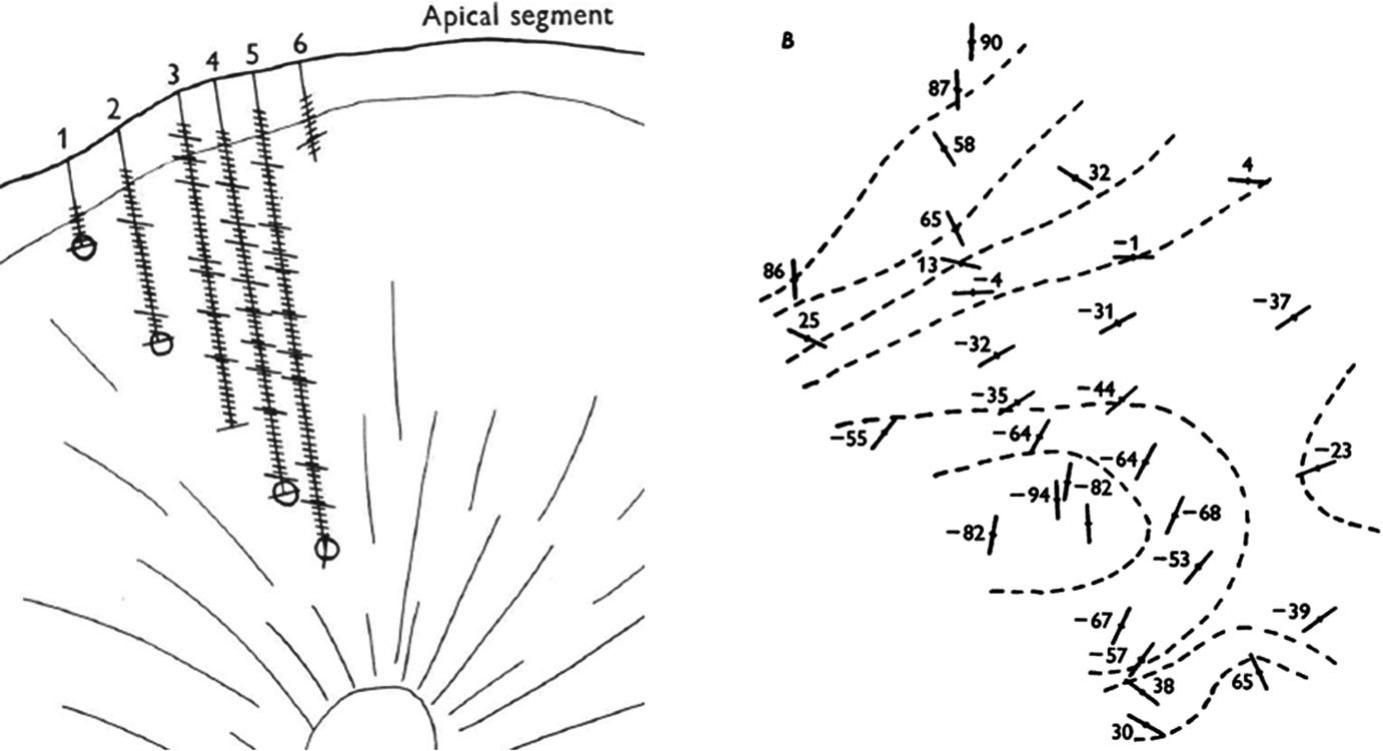
**a** Detail of Fig. 2 in Hubel and Wiesel (1963) showing six parallel penetrations in the cat post-lateral gyrus. Penetration 2–5 map out one orientation column, with the borders at lesions in 2 and 4 laterally, and 6 medially. Lesion 5 supposedly maps a column of the next section. **b** Surface map shows the distribution of similar orientations (ibid., Fig. 4b)

In their next study, Hubel and Wiesel (1963) used Hubel’s technique to produce electrolytic lesions to mark a shift in orientation along one electrode. A comparison of six closely spaced penetrations revealed that the lesions roughly fell in line with the radial fiber bundles that extended throughout the grey matter (Fig. 2a). But when Hubel and Wiesel returned from the deep recordings back to the surface, the three-dimensional shape of the columns barely resembled a pillar. Sometimes similarly oriented cells formed long bands that frequently took curved turns (Fig. 2b). Often they displayed even less of an order, so that Hubel and Wiesel could not describe a systematic arrangement of columns in the entire striate cortex (cf. ibid., 566).

The confusing arrangement of orientation columns was further complicated by the possibility that their boundaries were not discrete. At the distance of 50 µm, the technical limit of making adjacent penetrations, orientation shifts still occurred (cf. ibid., 562). Hubel and Wiesel had to rule out orientation shifts below this scale on conceptual grounds. Because orientation selectivity is a functional property of individual neurons, it cannot be truly continuously distributed.^7^ Again, the audiomonitor proved indispensable to decide the issue. Hubel and Wiesel frequently heard abrupt shifts in orientation of rather unpredictable size, sometimes as large as 45º or 90º. What appeared as a sign of disorder simultaneously counted as a proof of discreteness.

Whereas the column concept proved successful to describe orientation selectivity, it was initially of little use for investigating eye preferences of the visual neurons. Most cells showed mixed responses to inputs from the ipsilateral and contralateral parts of the visual field (cf. Hubel and Wiesel 1962, p. 125f.). Within an orientation column, cells of different eye preference groups intermingled, but short sequences suggested that “the cells could be arranged in nests, or conceivably in very narrow columns or thin layers” (ibid., 140). The evidential situation changed when Hubel and Wiesel (1965) produced artificial squinting in newborn kittens by cutting one muscle outside the eyeball. Because the resulting asymmetrical eye axes surprisingly had no striking effect on later visual performance, Hubel and Wiesel almost abandoned their project as failed (cf. Hubel and Wiesel 1998, p. 407). Eventually they decided to at least obtain one cortical recording, in which almost all cells were predominantly driven by one eye. With the new contrast observed in the deprived kittens, Hubel and Wiesel now re-examined their orientation surface maps from 1963 with regard to ocular dominance grouping. Patches with similar eye preference equaled the size of orientation columns, but were cross-cutting their boundaries. Were the ocular dominance columns arranged very narrowly as suggested in 1962, or 0.5 mm wide like Mountcastle’s columns, or did they span several millimeters (Hubel and Wiesel 1965, p. 1055)? All three options were feasible at this point. Since the penetrations in the surface maps were spaced too far apart to discern a finer pattern, Hubel and Wiesel decided for the larger scale by regrouping the results into “left” and “right” eye preference. Only the cells which were *equally* drivable by both eyes counted as cases of binocular interaction, whereas all other responses represented a continuum of increasing ocular dominance (cf. ibid., Fig. 9a).

When extending their work to primates (spider monkey and rhesus macaque) Hubel and Wiesel (1968) added the class of *hypercomplex* cells to their hierarchical wiring scheme. These neurons showed a decreasing or no response if stimuli extended beyond the activating region of the receptive field, which was typically surrounded by weak and strong antagonistic regions (cf. ibid., 220). While most cells in the monkey area 17 were more precisely tuned to orientation than in the cat, some cells surprisingly showed no orientation selectivity at all.^8^ In contrast to the cat, small orientation shifts in the monkey were arranged in 0.1–0.25 mm wide columns, but were also irregularly interrupted by large 45–90º shifts. Hubel and Wiesel countered these irregularities by resorting to an early recording in a spider monkey named “George” (cf. Hubel 1981, p. 39). Within one continuous 5-h penetration at a 30º surface angle, they encountered 53 orientation steps at 20 μm intervals, with two full 180º shifts in orientation axis. This order of magnitude implied that the functional columnar organization could actually correspond to the vertical cell rows seen in the histological sections (cf. Hubel and Wiesel 1968, p. 232). What seemed baffling was that the anatomical order seemed to prevail everywhere whereas the functional one did not, and where they coincided, the columns took the form of long and narrow swirling slabs, rather than straight cylindrical pillars.

Because cells in the monkey often belonged to the same or neighboring ocular dominance groups across several orientation columns, Hubel and Wiesel wondered whether columnar response properties might vary between *horizontal* layers. When they advanced to layer 4A, the cells showed simple receptive field responses that were monocularly driven. In layer 4B, cells frequently showed no orientation selectivity (cf. ibid., p. 235). Since field complexity and binocular interaction increased in layers 2/3 and 5 and 6, Hubel and Wiesel extended their putative wiring scheme to a feedforward model of processing *visual input*. Two columnar systems implemented the functions of analyzing contours and converging monocular input, with input at layer 4 cells and outputs occurring at the hypercomplex cells of the superficial layers. To account for binocular interaction, Hubel and Wiesel assumed a cross-connection of two neighboring ocular dominance columns. Different spatial relations to the smaller orientation columns were now possible: “a patchwork of alternating columns like a checkerboard, or a confluent matrix of one type with pillars of the other type embedded within it, or a series of parallel slabs” (ibid., p. 241).

**Fig. 3:**
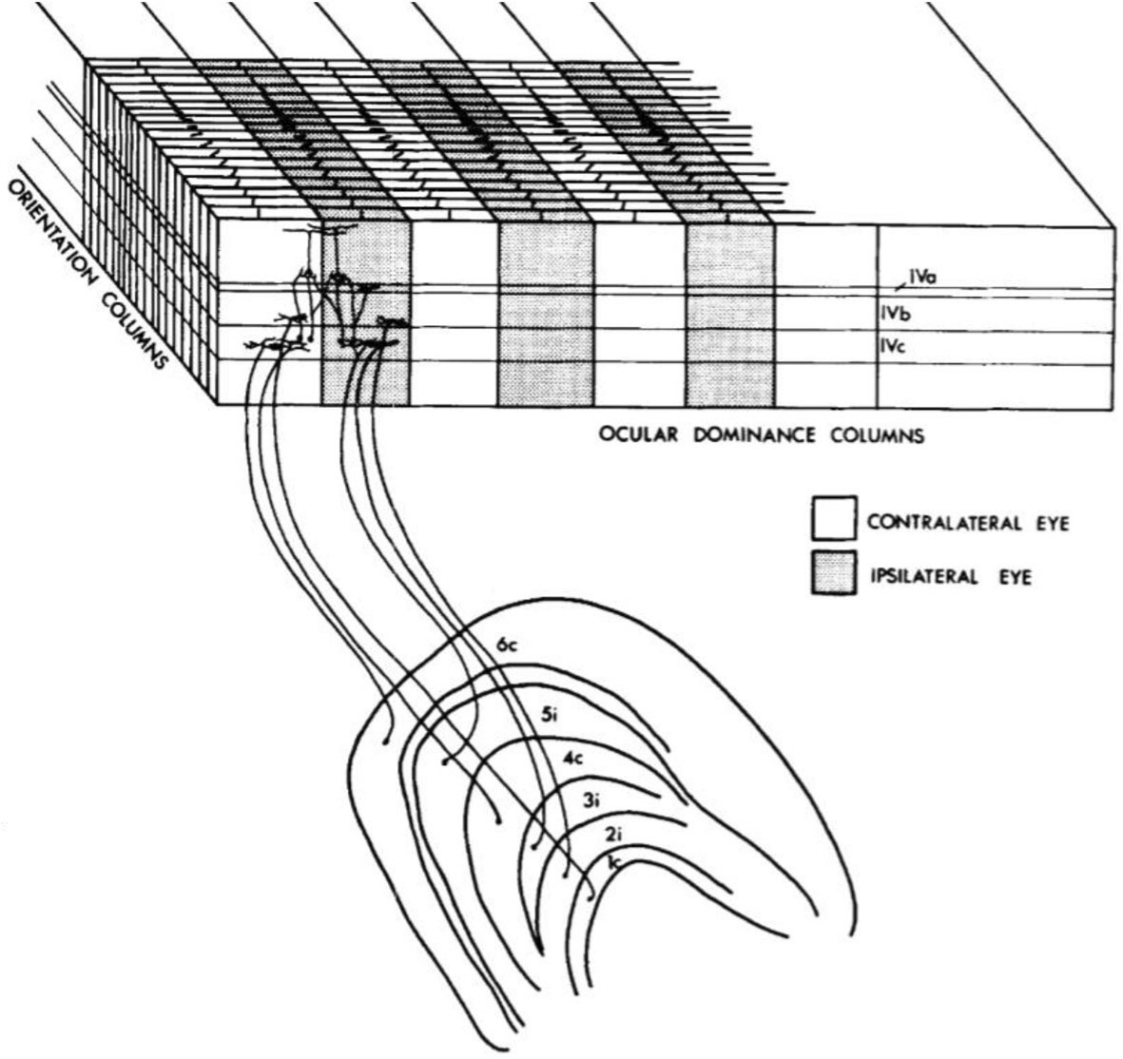
Hubel and Wiesel’s hierarchical wiring scheme (1972), Fig. 18, p. 449). Monocular LGN cells connect to simple layer IVc cells in different ocular dominance columns, which cross-connect to complex cells in higher layers. Orientation columns are stacked orthogonally to this arrangement

In order to decide between the different possibilities of columnar structure, Hubel and Wiesel (1969, 1972) introduced a modified version of the Nauta silver-impregnation method that visualizes both degenerating axons and their synaptic termination points (boutons). They suspected that lesioning a monocular LGN layer should produce a distinct degeneration pattern in cortical layer 4. Therefore, both structures were silver-stained and sometimes Nissl-counterstained to determine the layer locations. When lesioning the dorsal and ventral geniculate layers individually, the stained boutons were distributed in stripes that were separated by terminal-free interbands. Whereas the staining of axon fibers alone would have not revealed this column-like pattern, the 0.25–0.5 mm wide bouton stripes now suggested that the ocular dominance columns had a slab-like structure.^9^ But since the slabs were much wider than the orientation columns, how could a binocularly driven complex cell in the same orientation column receive inputs from two different ocular dominance columns? Hubel and Wiesel’s solution reconceived the two column structures as *orthogonally* positioned to one another. They presented the result in a diagrammatic wiring scheme, which provided the first prototype of what became later known as the “ice-cube model” of visual cortex (Fig. 3).

Since the 1961 recording from monkey George was the only evidence for ordered orientation sequences, Hubel and Wiesel (1974a) adjusted their experimental procedure to investigate the supposed geometrical order of orientation columns. Electrodes with coarser tips and lower impedance enabled continuous recordings, and tangential penetrations promised to cover several vertical orientation columns in one long-distance recording (cf. ibid., 268). In one paradigmatic penetration, Hubel and Wiesel observed discrete 10º orientation shifts every time the electrode advanced 25 to 30 µm.^10^ The result left open, however, whether the discrete steps were only an artifact of the coarse tips sticking to the tissue. Whether a finer orientation tuning existed below this limit of resolution was indeterminate. Like in 1963, Hubel and Wiesel argued that orientation shifts were already close to singe cell spacing. Unfortunately, in the histological reconstructions, they counted 32 Nissl-stained cell bands in a sequence of 22 orientation shifts (cf. ibid., p. 271). Because they could not decisively settle the question of continuity or discreteness, Hubel and Wiesel had to admit that “either one must broaden the definition of the column or decide that the [orientation] system is not strictly columnar” (Hubel and Wiesel 1974a, p. 289). The situation had not substantially changed since their first experiments in the cat, except that the sequential order of small regular orientation shifts now presented “the rule rather than the exception” (ibid., p. 268).

Finally, Hubel and Wiesel (1974a) provided a general application criterion for the column concept: A spatiotemporal region of cortex counts as a column if there exists a vertical subdivision with cells lining up perpendicular to the surface and layers. In an ideally perpendicular penetration into such a structure, the recorded functional cell properties (here: visual field position, orientation and ocular dominance) would remain virtually constant. It is notable that Hubel and Wiesel’s criterion did not specify whether cortical columns were literally shaped like a pillar, or were organized into a more complicated geometrical structure. After almost two decades of research, the evidential landscape was still confusing. Even the stripe-like ocular dominance columns frequently occurred in an interlacing pattern. For the slab-like but curved pattern of orientation columns, the direction of their arrangement in the cortex remained entirely unclear.^11^ As in earlier papers, the columnar order had to be justified conceptually by the *principle of economic wiring*. The present knowledge about thalamic cortical input suggested that juxtaposing similarly oriented cells along their eye-preferences minimized the wiring costs between connected cells.

### The conceptual outlook of the cortical column

Between 1958 and 1974, Hubel and Wiesel applied new anatomical techniques and refined Mountcastle’s physiological recording methods to turn the cortical column into a more determinate research object. The column now appeared as the central concept to describe the visual cortex as being organized into simple, complex and hypercomplex cells with similar receptive field orientations, and as segregated into bands and interbands according to a wiring scheme that fulfilled the principle of economic wiring. I propose to call this position, from which researchers like Hubel and Wiesel started their experimental investigations, the *conceptual outlook of the column.*

The term “conceptual outlook” is used by Lange (2000) to describe how different conceptual assumptions about a system make different patterns in a data set salient.^12^ In the case of the column, vertically similar response properties would not have been salient from a viewpoint centering on the horizontal layers of the cortex. The conceptual assumption of functionally significant vertical cell bands then allowed Hubel and Wiesel to use several inductive strategies to anticipate columnar patterns in the recorded data. Like Mountcastle, they could predict variances in single cell responses in relation to the electrode angle, and attribute a shift in response properties along one electrode to distinct columns if the true axis to the surface was known (Powell and Mountcastle 1959; Hubel and Wiesel 1963). New strategies like the diagrammatic plotting of orientation shifts also allowed Hubel and Wiesel to predict upcoming data values, because they continued an orderly orientation pattern known from the monkey “George” recording. Columnar patterns often became salient in combination with other concepts: in light of the hierarchical wiring scheme, for instance, the arrangement into orientation columns took on “new meaning” (Hubel and Wiesel 1962, p. 144). The scheme furthermore implied anatomical connectivity patterns that the researchers could search for with anatomical techniques (Hubel and Wiesel 1968, p. 241).

A descriptively adequate account in philosophy of neuroscience (Craver 2007) should answer why the term “columnar outlook” describes Hubel and Wiesel’s experimental practice more appropriately than the terms “columnar theory” or “columnar hypothesis”. I think that a case against “theory” can be made because Hubel and Wiesel studied functional properties of neurons—like orientation selectivity—that had simply been unknown so far, and could therefore not yet be part of any theory. The experiments on the organization of such properties were *exploratory* and did not test a specific theory about how the visual cortex works (see also Hubel and Wiesel 1998, p. 411). One exploratory research strategy is exemplified by Hubel and Wiesel’s use of different stimuli such as light and dark spots of different shape and size, or different stimulus orientation with respect to the eye field. By systematically varying these experimental parameters they found a *general pattern* in their data (see Steinle 1997), i.e. that cells of the same orientation or eye-preference are vertically grouped in the cortex. The use of the column concept to describe and anticipate this pattern did not comprise or make any reference to a theory.^13^ These investigations rather combined different experimental techniques (see also Burian 2001): Hubel’s eye stimulations manipulated visual processes at the system level, while Wiesel’s electrode recordings measured cortical responses at the component level. Together with the column concept and anatomical background assumptions, the exploratory strategies helped Hubel and Wiesel to inductively infer columnar response patterns in unobserved cases without necessarily developing a specific theory of the visual cortex.

So while the columnar outlook thus described is less specific than a theory, it is also much broader than a particular hypothesis. By “hypothesis” I mean a factual statement that is derived from a theory in advance of, and can be falsified through a requisite experiment (see Glass and Hall 2008). According to this working definition, I claim that Hubel and Wiesel did not test the “columnar hypothesis”; they rather used the column concept to *formulate* more particular hypotheses. Two examples of such hypotheses would be that a lesion in geniculate layer should produce a columnar anatomical pattern in parts of the cortex (Hubel and Wiesel 1972), or that closely spaced parallel penetrations will record similarly oriented cells of the same column (Hubel and Wiesel 1963). While these particular hypotheses can be true or false, the concept of the “column” cannot be true or false. Concepts can be only *appropriate* or *inappropriate* to describe entities in the domain of investigation (cf. Steinle 2009, p. 120). Another way of thinking about the difference is to view the column concept as part of a “style of reasoning” (Hacking 1983) that determines what kind of empirical claims *can* be true or false at all. The columnar outlook therefore corresponds closely to Hubel and Wiesel’s “laboratory style of thinking and doing” (Hacking 2007) that determines which statements about mesoscopic functional architecture can be candidates for verification or falsification. Refuting a specific hypothesis about columns therefore does not require the researchers to abandon the concept altogether: although Hubel and Wiesel refuted Mountcastle’s hypothesis that columns are pillar-shaped, they could appropriately describe their results by reconsidering the structure as swirling slabs.^14^

Hubel and Wiesel’s move from pillars to slabs also illustrates how extending the application domain of a scientific concept often necessitates revisions that accommodate for evidence that conflicts with the concept’s initial definition. Hubel and Wiesel did not deem exact geometrical concordance of structure as a *necessary* feature of different kinds of columns, as long as vertical subdivisions were present. Moreover, the columnar outlook proved fruitful exactly because columnar response patterns could be *differentially reproduced* if a new element was introduced into the experimental system (cf. Rheinberger 1997, p. 89f.). These elements included different animal models (cat, spider monkey, macaque), experimental stimuli (skin, eye), or recording technologies (Davies chamber, tungsten electrodes, audiomonitor). Subsequent changes in all three categories even led to a borderline case of applying the column concept: it became indeterminable whether gradual orientation shifts during a continuous electrode recording should still be counted as a response pattern of discrete columns (Hubel and Wiesel 1974a). Either the column definition had to be extended to continuous variables or the column concept was altogether *inappropriate* to describe this functional feature of mesoscopic architecture.

To see the difference between such a conceptual change within the columnar outlook and within a supposed “columnar theory”, consider Hubel and Wiesel’s remark that counting gradual orientation shifts as “columnar” is “a matter of taste and semantics” (Hubel and Wiesel 1974a, p. 289). It sounds as if accommodating for these results amounts to a (merely optional) shift of semantic relationships in the theoretical framework surrounding the column concept. But I urge to resist the notion of a “columnar theory” that was evaluated independently of what happened in Mountcastle’s or Hubel and Wiesel’s experiments. In contrast to such an independent theory, the columnar outlook made continuations of response patterns salient because it relied directly on the *experimental strategies* that Hubel and Wiesel used in their exploration of the visual cortex. Instead of keeping separate scores of a set of general facts about columns on the one hand, and experimental results in a particular system on the other, the columnar outlook and the kinds of experiments discussed above were of a piece. Therefore, I propose that the issue is not merely whether we call gradual orientation shifts “columnar”, but whether it makes sense to investigate the *behavior* of visual cortical neurons with perpendicular electrode penetrations or not. Consequently, it is the behavior of the entities *themselves* that initiates conceptual change (cf. Haugeland 2013, p. 58f.). Determining the features and application domain of the column concept is not a matter of taste and semantics, as Hubel and Wiesel surmised, but a matter of “getting the entities right” (ibid., p. 199).

I think that the experiments described above show that Hubel and Wiesel actually did try to get the entities of the visual cortex “right”, because all experimental procedures were increasingly and exclusively focused on the behavior of the column.^15^ The Davies recording chamber and the tungsten microelectrode were used to record columnar response patterns and to produce electrolytic lesions that made the columnar structure traceable in the histological sections. Moving spot stimuli were used to map the receptive field properties of orientation and ocular dominance columns. The sounds of the audiomonitor played a central role in connecting the space between two isolated units, and to tell apart their orientations or eye input (cf. Hubel and Wiesel 1962, p. 129). Hubel worked the projection screen with varying visual stimuli, while Wiesel recorded the cells to map their receptive fields on paper. In the midst of this configuration, the anesthetized cat in a head-holder faced the projection screen. The only elements “acting freely” in this experimental system were the cortical neurons—at least within the range of optimal responses. The major limiting factor was therefore not unreliable instruments, disruptive animal behavior or fading cell activity, but *human fatigue*. Hubel and Wiesel later contrasted this “style of thinking and doing” with the “style of working in awake, behaving animals [which] involves recording not in stints of 24–36 h, or till one collapses from exhaustion, but just for a few hours a day, or until the monkey gets fed up with fruit juice.” (Hubel and Wiesel 1998, p. 411).^16^

To summarize, I argued in this section that from the columnar outlook, researchers were able to pursue various inductive strategies and to formulate specific hypotheses about the functional organization of the cortex. I illustrated the difference between an independent theory and the columnar outlook with the issue of continuous functional transitions between orientation columns. Because the columnar outlook is closely tied to the experimental conditions, I proposed that conceptual change resulted when the neurons’ *behavior* violated the expected form or continuation of the columnar response patterns. In the last section, I show that the columnar outlook briefly took the form of a “general theory”, before new experimental techniques started to diversify column research.

## The internal articulation of the columnar outlook

### Hypercolumns and the ice cube model of the visual cortex (1974–1977)

The last stage of early column research led to a refinement and extension of the columnar outlook. It began when Hubel and Wiesel (1974b) introduced a new concept that both referred to a columnar superstructure and subsumed all their previous findings. What was unknown so far was how column size remained constant while receptive field sizes increased with the visual field degrees covered by 1 mm of cortex. Hubel and Wiesel used this inversion of the so-called “magnification factor” to assess how the random scatter relates to the increasing size of the receptive fields. Five recording locations between 1° and 22° eccentricity from the fovea suggested that inverse magnification and receptive field size change roughly in a parallel fashion (cf. ibid., 301). Given that a 180° shift in orientation also occurs about every 0.5–1 mm, Hubel and Wiesel tried to capture this uniformity conceptually by introducing the *hypercolumn*, which contains a full set of orientation columns and one left and right ocular dominance column. They inferred from the dimensions of the hypercolumn that “a 2–3 mm region of cortex can be said to contain by a comfortable margin the machinery it needs to analyze the region of visual field that it subserves” (ibid., 303). The systematic link between receptive field scatter and magnification finally solved the puzzle of how visual topography related to columnar organization. Because the column boundaries lie below visual field changes, Hubel and Wiesel inferred that visual input must be processed (almost) uniformly throughout the entire primary visual cortex.

In the Ferrier lecture held in 1972, Hubel and Wiesel (1977) finally related their findings explicitly to the “first five or six steps in the processing of visual information” (ibid., 5). By adding the concept of an *aggregate receptive field* to their conceptual outlook, mean field coverage at a vertical penetration became more salient than the random scatter in position (cf. ibid., 14). Picking layer 3 as an empirical example of moderate field size and scatter, Hubel and Wiesel concluded that “the overall law is that for this layer a 1–2 mm displacement along the cortex is about enough, on the average, to displace the aggregate field into an entirely new terrain” (ibid., 14). They further argued that the two column systems can solve the task of “analyzing the building blocks of perception” (ibid., 17) within this 2 × 2 mm block of tissue. Nevertheless, they also admitted that the functional purpose of the ocular dominance columns was actually unclear. It was clear that these columns in area 17 achieved partial mixing of eye influences, but whether area 18 used this mixed input to build up stereoscopic depth perception was nothing but educated speculation (cf. ibid., 21). The indeterminacy of function was especially perplexing given that the ocular dominance columns were Hubel and Wiesel’s most stable anatomical finding. In contrast, orientation columns were functionally well-described but their actual anatomical connectivity pattern was entirely unknown.

**Fig. 4:**
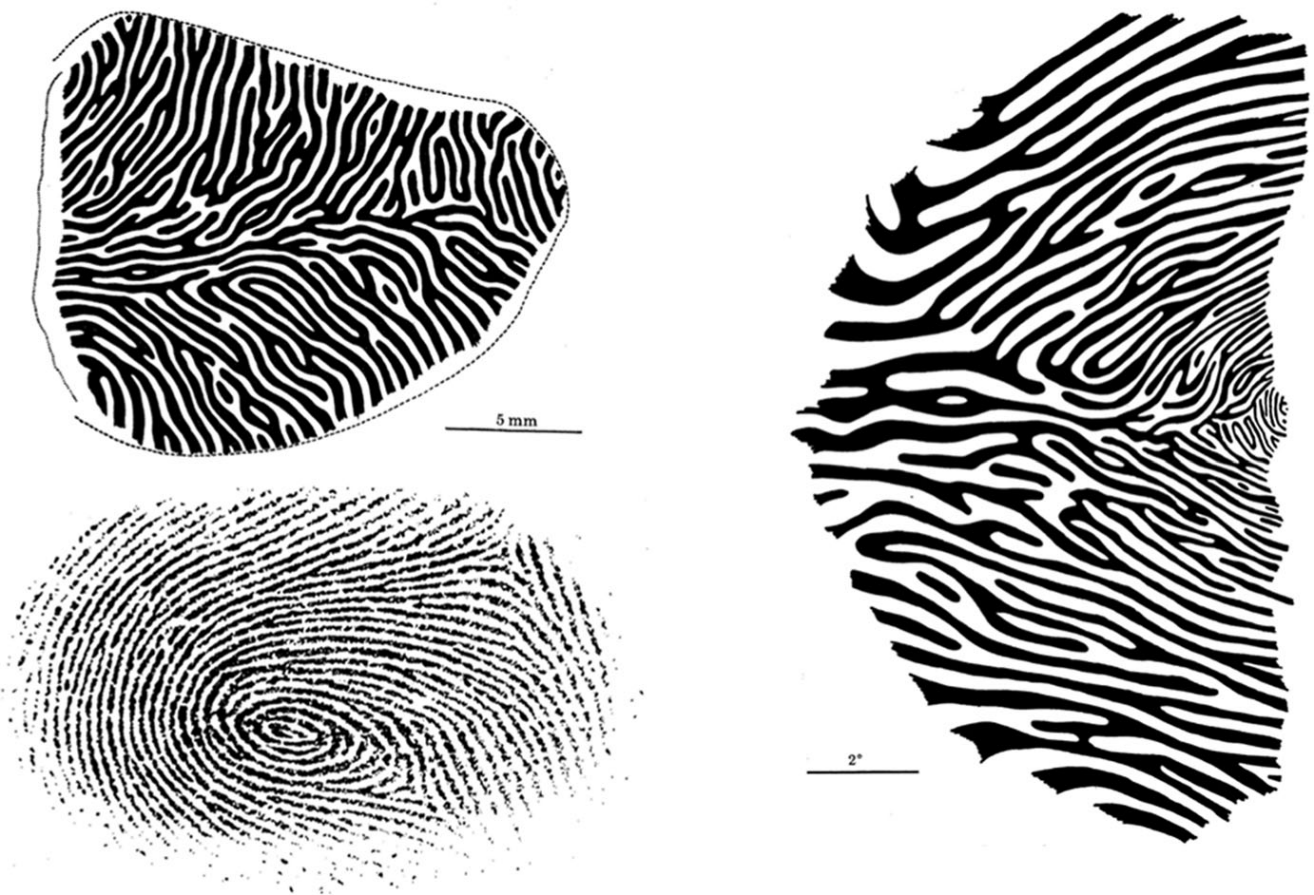
**a** *Top* shows reconstruction of entire layer IVc ocular dominance column (right hemisphere). *Bottom*: Hubel’s fingerprint to compare the scale (from Hubel and Wiesel 1977, p. 35). **b** Reconstruction of (**a**) translated into the left visual field. Ocular dominance patterns are mapped from fovea (*right*) to 9º eccentricity (cf. ibid., 36)

When using reduced silver staining to find an anatomical counterpart of orientation columns, for instance, Hubel and Wiesel’s anatomist Simon LeVay instead saw dark stripes that were 400 µm wide and ran parallel through the upper part of layer 4c (cf. ibid., 32). Since the stripes occasionally joined where eye inputs were expected to converge, the researchers concluded that they must represent the ocular dominance columns. However, a major drawback of this method for large reconstructions was the need to cut the tissue tangentially to layer 4 to see silver stains in the sections. Autoradiography presented an advantage because the connections did not have to be inferred, but were directly stained by radioactive amino acids injected into the eye, which are axonally transported to the cortical termination site. The silver grains that bound to the deposited amino acids in layer 4c again showed 0.4 mm patches characteristic of ocular dominance columns.^17^ To synthesize their autoradiographic data, Hubel and Wiesel cut layer IVc such that it had the shape of a ring with increasing diameters. The superimposition of rings would then give a reconstruction of the columnar pattern of the entire layer IV in the exposed part of the visual cortex. To show the scale of the pattern, the researchers put Hubel’s index fingerprint next to the reconstructed image (Fig. 4a).

What is striking is how the projection of the anatomical pattern into the visual field (Fig. 4b) looks like one half of a “visual fingerprint”. The analogy to fingerprints captures the observation that columnar structure is seen already in newborn monkeys and presumably does not change during lifetime (cf. ibid., p. 43ff.). Thus, the columnar pattern constitutes an individually specific feature that nevertheless follows certain species-general rules (cf. ibid, p. 34, where the pattern is compared to zebra stripes). Besides the analogy between the fingerprint and the ocular dominance pattern, the choice of Hubel’s index finger may also indicate a personal relationship to the research object. It appears as if Hubel’s fingerprint is supposed to unambiguously identify the scientists at the scene of anatomical discovery.^18^ This interpretation is also supported by the fact that Hubel and Wiesel heavily stressed the importance of anatomy for the credibility of their electrophysiological experiments (cf. Hubel 1981, p. 37). The choice of the fingerprint as analogy and personal mark shows the significance of the ocular dominance columns within the columnar outlook, even though their biological function remained largely elusive.^19^

With the full picture of ocular dominance structure, Hubel and Wiesel could now return to the idea of the hypercolumn as a recurrent superstructure. If a penetration started at the hypercolumn boundaries, the cells within this structure should be able to analyze the entire visual field input for edges at every orientation. Based on this criterion of applying hypercolumns, Hubel and Wiesel introduced the *ice cube model of the visual cortex* as an abstract and idealized version of the data about columnar response patterns they had accumulated. Since philosophers such as Bechtel (2001, 2009) often discuss Hubel and Wiesel’s work in the context of discovering the neural mechanisms of visual perception, it is tempting to view the ice-cube model as a *mechanistic* model (see Craver 2006). Besides the lack of proper mechanistic decomposition in electrophysiological studies (Sect. 2.1), I think that the ice-cube model also does not add any explanatorily relevant information that would go beyond the basic mechanism sketch embodied in Hubel and Wiesel’s wiring scheme of 1962. Bechtel (2009, p. 547, 552) rightly describes Hubel and Wiesel’s research as the starting point for the mechanistic decomposition of vision, mainly because they (tentatively) explained how LGN input is transformed into orientation-selective output by simple and complex cells. But that they discovered that these cells were organized into *columns* is only mentioned in passing (Bechtel 2001, p. 232). Rather than adding explanatory detail, the ice-cube model provided a concise *description* of 15 years of experiments. It suggested that aggregates of the same mechanism form recurrent units for the analysis of the “building blocks of vision” (Hubel and Wiesel 1977, p. 45). I therefore claim that the achievement of the ice-cube model was not that it increased explanatory power, but that it put Hubel and Wiesel’s columnar outlook into one (deceivingly simple) picture, to be reprinted in neuroscience textbooks of decades to come.

### The columnar outlook as a general theory of the cortex (1978–1981)

Building on Hubel and Wiesel’s idealized model of the visual cortex, Mountcastle (1978) extended the columnar outlook to the entire cortex, such that it appeared to provide a theory of the brain as a dynamic information processing machine with a distributed architecture. He proposed that macroscopic brain areas that implement cognitive functions are composed of cortical columns—modules of information processing that invariantly compute outputs from the area-specific inputs across the entire cortex. By inferring a canonical architecture from cat and monkey experiments, Mountcastle defined columns in the human brain not in terms of the cognitive functions they served: “there is nothing intrinsically motor about the motor cortex, nor sensory about the sensory cortex” (ibid., 9). His statement was justified anatomically by the observation that neuron number within small cylinders of a 30 µm section remain basically constant at 110 cells throughout the entire cortex (Rockel et al. 1974).^20^ The idea of the column as a uniform anatomical module was further supported by developmental studies, showing that cells migrating from the neural tube are guided by radially oriented glial cells (Rakic 1974). The resulting vertical cell bands were commonly found in the anatomical sections of the column experiments discussed above. Mountcastle (1978) now specified six region-independent criteria of what makes a column *a column* (cf. ibid., 16). Accordingly, a cortical column is an (i) input–output processing device which (ii) maps functional variables onto the cortical surface. By virtue of its internal connectivity, (iii) topological relations of the input to a column can be preserved across brain areas. The (iv) variation of response properties and the (v) partial topographic overlap between columns allows single columns to be recruited by an inhibitory mechanism. Finally, (vi) heterogeneous signaling pathways enable feature-selective processing of incoming complex stimuli. Unlike Hubel and Wiesel’s ice cube model, the purpose of Mountcastle’s list was not to derive hypotheses about one specific system but to assess whether a set of electrophysiological data from a cortical area qualifies as belonging to the columnar domain.^21^

Mountcastle’s attempt to unify electrophysiological research in five cortical systems under the generalized notion of the column suggests that he aimed to formulate a general theory of cortical processing. Needless to say, his proposal did not stand the test of time, since most neuroscientists today would dispute that the cortex is composed of, or operates through, uniform vertical cell bands (see, for instance, Swindale 1990, Horton and Adams 2005, Rakic 2008). In the remainder of this section, I mention three strands of the diversifying column research to explain why Mountcastle’s unifying theory of columnar modularity did not have a lasting impact.

Firstly, an alternative to Mountcastles proposal was that the principle of topographic representation was independent of cortical modularity, at least in the visual system (see also Creutzfeldt 1983, p. 414). Even though the ocular dominance columns were mapped below mean shifts in visual field position, their shape directly depended on the spatial distribution of the afferent fibers coming from the retina. They closely resemble Mountcastle’s deep and skin columns in that respect (Hubel and Wiesel 1972, p. 42). But the resemblance is at odds with the claim that the hand and feet areas in the somatosensory cortex are the counterpart to the alternating eye inputs of the visual columns (cf. Mountcastle 1978, p. 23). From Hubel and Wiesel’s columnar outlook this comparison did not make sense because the cortical topography of the visual field “does not by itself constitute a columnar system” (Hubel and Wiesel 1968, p. 230).

Secondly, when Mountcastle et al. (1975) investigated columns in behaving monkeys, they frequently observed optimally responding cells that were scattered throughout columns with cells that had passive and stable response properties. According to the researchers, the dynamic superimposition of these “‘out of place’ cells upon the columnar organization of areas 5 and 7 were observed too frequently, we believe, to be dismissed as due to experimental error” (cf. ibid., 903). The ability to robustly produce such response patterns even led them to postulate cells that were organized independently from the columns. Perhaps, then, Mountcastle’s introduction of the concept of a *cortical minicolumn* was more relevant than the ambiguous behavioral data (Mountcastle 1978, p. 37). It resolved the difference in order of magnitude between functional response properties (0.5–1 mm) and structural columnar patterns (25—50 µm). As argued in Sect. 3, the only cell-scale columnar response properties were orientation shifts, whose discrete or continuous distribution remained indeterminate. Thus, no unambiguous functional evidence existed for the claim that minicolumns make up the cortical machinery within one column.

Thirdly, by the time Hubel and Wiesel received the Nobel Prize for physiology and medicine (with Roger Sperry) in 1981, the solidified columnar understanding of the cortex was already outpaced by new experimental findings. In particular, the technique of staining with cytochrome oxidase (an enzyme indicating increased neuronal activity; see Wong-Riley 1979) had “opened up a system whose existence we were previously quite unaware of and whose anatomy and functions we do not yet understand” (Hubel 1981, p. 53). Like in the case of Mountcastle et al. (1975), the new unknowns arose when Kennedy et al. (1976) let the unanesthesized monkey have a look around in the laboratory. In his PhD thesis, Hubel and Wiesel’s student (and later column critic) Horton (1984) showed that cytochrome oxidase patches coincided with every second dot of Kennedy’s deoxyglucose pattern after monocular stimulation of the monkey striate cortex along all orientations. The finding implied that the cells at the intersection were either not orientation selective, contained a mix of different orientations, or collectively tuned for all orientations such that this variable would factor out of the deoxyglucose activity levels (cf. ibid., 233). Drawing on the theoretical findings of Braitenberg and Braitenberg (1979), Horton even went so far as to call the cytochrome blobs the fundamental cytoarchitectonic unit that *generated* the orientation selectivity of the surrounding cells. Because the blobs were centered in the ocular dominance columns, they provided the anatomical cornerstone of the arbitrary hypercolumn boundaries. Therefore, the insertion of the blobs into the ice-cubel model (Horton 1984, p. 249, Fig. 49) can be read as another case where new entities and their unexpected behavior forced the researchers to revise the columnar outlook.

## Conclusion

In this paper, I analyzed early experiments on the cortical column to argue that this concept opened up a new domain of inquiry in 1955, because it captured a previously unknown phenomenon under laboratory conditions. I followed the development of the column concept until 1981 by arguing that the experimental and technological conditions entrenched this concept by offering application criteria that could be used in further electrophysiological experiments. Hubel and Wiesel’s move from a pillar-shaped to a slab-shaped view of columns in the visual system is an example of a conceptual revision resulting from changed experimental conditions. I furthermore rejected the idea that the new columnar understanding constituted a specific theory of the (visual) cortex. It rather provided a broader outlook on the functional architecture of the cortex, on the basis of which researchers could anticipate unobserved columnar response patterns from previous (concept-laden) observations. By showing how the columnar outlook depended on Hubel and Wiesel’s experimental system, I also argued that conceptual revisions were induced by the unanticipated behavior of the entities themselves, rather than simply by the semantic decisions of the researchers. In the final part of the paper, I showed how the columnar outlook became embodied in Hubel and Wiesel’s ice cube model, and how it led to Mountcastle’s general theory of columnar modularity.

The analysis of early column research presented here can be seen as the initial part of a larger project that traces the status of the column as an organizational unit of the cortex from its discovery to the present. I already indicated at the end of the paper that the next stage of this history is characterized by a proliferation of techniques like cytochrome staining that led to results conflicting with the classical columnar outlook. Optical imaging also recorded unoriented cells in “pin-wheel centers”, but a clear correspondence to cytochrome oxidase patches could not be established. Computer models furthermore converged on simulating orientation as a dynamic network rather than a stable cell property. Mountcastle’s general definition of the column also became increasingly questionable with the rise of comparative anatomical studies, which discovered species that lack columnar structures but possess the corresponding functions. The developmental mechanism postulated by the radial unit hypothesis of cortical formation was subsequently used by Horton and Adams (2005) to claim that the vertical cell bands may be a functionally insignificant by-product of ontogenetic development.

 A full history of the column would finally map the different research strategies that are possible today, after the proclaimed “death” of the column by Horton and Adams (for a similar treatment of the concept of a “receptive field”, see Chirimuuta and Gold 2009, p. 212ff.). I previously sketched three research strategies that assign a different status to the column concept (Haueis 2014a). Firstly, researchers can apply the column concept to locally variable structures without assuming an underlying uniformity of the cortex (T’so et al. 2009). Secondly, based on their connectivity, columns can be redefined as parts of distributed networks that can be investigated with novel neuroanatomical tracing methods and multi-electrode recordings (Rockland 2010). Thirdly, the “canonical microcircuit” could replace the column as a basic unit of mesoscopic brain organization without assuming a vertical anatomical module (da Costa and Martin 2010). Depending on which strategy is adopted, different implications follow for the use of the column concept in contemporary approaches to brain organization, such as connectomics (Sporns et al. 2005) or computer simulations (Markram et al. 2015).

## Footnotes

1 The word “remains” is essential here: at the time of their introduction, none of these concepts was unanimously accepted as describing basic brain organization (cf. Jacobson 1993, chap. 3; Mountcastle 2009, p. 351).

2 As a result of my historical restriction to column research until circa 1981, I do not discuss later but relevant papers here. Da Costa and Martin (2010) provide an abbreviated reconstruction of the material discussed in Sects. 2.1 and 3.1, although with the goal of replacing the notion of the column with that of the “canonical microcircuit”. This concept poses its own issues and questions, which cannot be adequately analyzed within the scope of this paper. More recently, researchers from the “Human Brain Project” reconstructed and simulated what appears to be a column within the rat somatosensory cortex (Markram et al. 2015). Although a further analysis will not be given here, it should be noted that these researchers use the term “microcircuit”, presumably because hind-limb somatosensory cortex has “no anatomically defined horizontal columnar organization” (ibid., 462).

3 Mountcastle admitted that their method was not unbiased since anesthestic effects and the electrode form neglected weakly active and small neurons respectively (cf. ibid., p. 402). Additionally, the researchers could not say with certainty which cell they were recording from. Similar amplitudes over a vertical distance of approximately 100 µm (cf. ibid., 404) indicated that the electric field of any recorded neuron influences and is influenced by hundreds of other cell bodies within this range.

4 Note that Lorente de Nó, following Rose (1912), mislabeled this structure as the mouse acoustic cortex, and that the rodent cortex also does not contain vertical columns as Mountcastle’s defined them (cf. da Costa and Martin 2010, p. 1, 7). De Nó’s vertical unit therefore does not straightforwardly support Mountcastle’s definition empirically, but rather resembles it in some conceptual features.

5 While a scientific understanding of these material parts of the experimental design is required, the theory about, say, the microelectrode will not predict or explain why there is a columnar response pattern in the data (except if it is an artifact of the recording process). See also Hacking (1983, p. 182).

6 Hubel’s recordings from chronically implanted electrodes were significant for three reasons. Firstly, they showed that the absent response of cat cortical neurons to diffuse light could not be attributed to anesthetic effects or altered physiological parameters (temperature or blood pressure; cf. Hubel 1959, p. 233). Secondly, they ruled out that the ineffectiveness of Kuffler’s retina stimuli (large light spots) in the cortex were induced by anesthesia (cf. Hubel and Wiesel 1959, p. 586). Thirdly, they established direction-dependent movement as an important stimulus parameter, which the researchers later used to map out receptive field axis and ocular dominance (cf. Hubel and Wiesel 1962, p. 112f.).

7 Notice that this argument becomes somewhat trivial if it is acknowledged that a neuronal representation of functional properties is never actually continuous, because neurons are discrete entities. However, Hubel and Wiesel’s argument for discreteness can also be understood as a practical remark about their measurements: if two orientation shifts are sufficiently close together, there cannot be a third measurement that records an orientation value that lies between the two.

8 The cells furthermore turned out to be less color-sensitive than expected from previous LGN recordings, and their sensitivity did not clearly relate to their complexity type (cf. ibid., p. 225).

9 Hubel and Wiesel primarily relied on bouton distributions, presumably because the degenerating axons alone gave not a clear indication of the width of ocular dominance patches in the anatomical sections (cf. Hubel and Wiesel 1972, Fig. 2, p.  426f. and Fig. 3c, d, p. 429).

10 To alleviate the measurement error of 2º–5º, the researchers graphically plotted distance against orientation to anticipate the next value from the slope of the previous data points. Curves of the tangential penetrations were regular and their steep slopes produced the expected contrast to the perpendicular recordings (cf. ibid., p. 269).

11 The intracortical connectivity of orientation columns could not be determined anatomically because the available silver staining methods could only track degenerate extracortical connections. What was even worse, dendritic and axonal arborizations seen in Golgi sections extended way beyond the estimated column boundaries. To explain away this discrepancy, Hubel and Wiesel had to postulate inhibitory connections between neighboring columns, for which no physiological evidence existed (cf. Hubel and Wiesel 1974a, p. 293).

12 Lange illustrates his idea with the van der Waals law in chemistry. The law makes an increase of the product between pressure and volume of gases salient if scientists assume that gases consist of small moving particles that will be so close together at high pressures that the volume will decrease more slowly than the pressure will rise (cf. Lange 2000, p. 219ff.). What prima facie looks like a discontinuous series of gas states, presents itself as the continued inductive confirmation of the van der Waals law from the ‘molecular-gas’ outlook.

13 In contrast, psychophysical channel theory has used the concept of orientation selectivity since 1969 to explain how the visual system analyzes images by breaking them down into Fourier components (cf. Chirimuuta and Gold 2009, p. 205). But as far as I can see, the channel theory played no role in the electrophysiological and anatomical investigation of orientation columns.

14 The distinction can be illustrated by the example of the rodent visual cortex, which lacks orientation columns (Horton and Adams 2005, p. 837). In order to empirically determine whether the hypothesis that “orientation-selectivity in rodents is organized into columns” is true or false, researchers need to accept Mountcastle’s and Hubel and Wiesel’s experimental techniques as providing evidence for the behavior of cortical neurons. Since these techniques offer the application criteria for the column concept, the concept is already presupposed when formulating the hypothesis to be tested, rather than being tested itself. This is what is meant by saying that the column concept—and the experimental practice that accompanies it—opens up the possibility for particular hypotheses to be true or false.

15 My point is that earlier experiments were not—or at least not so exclusively—designed to investigate columnar organization (e.g., Hubel and Wiesel 1959; Hubel and Wiesel 1962).

16 Hubel and Wiesel’s quote also illustrates how the environment of the scientists was shaped by the material configuration of working with electrophysiological instruments on anesthesized animals. One might therefore call their experimental system an “experimental microworld” for the column (cf. Rouse 1987). This concept emphasizes that Hubel and Wiesel’s working environment also provided a system in which cortical neurons showed an orderly behavior under isolated circumstances that were carefully controlled by the experimenters. Continuing the metaphorical diction of the paper’s title, one could say that the cortical column “lived” in the experimental microworld that Hubel and Wiesel set up to work with it, and that, within this microworld, it showed the behavior leading to the classical columnar outlook.

17 Note that the subsequent use of autoradiography by Hubel, Wiesel and Stryker (1978) did not elucidate the connectivity pattern of orientation columns either. What this study found were 570 µm wide stripes in monkeys that were visually stimulated, i.e. patterns of orientation *hypercolumns*, not patterns of neurons that are axonally connected within and across individual orientation columns (cf. ibid., p. 371, and Fig. 8a).

18 For a discussion of the use of fingerprints in art images (“Täterbilder”), see Uppenkamp (2010). For the status of the first automated fingerprint systems for identifying individuals at the time Hubel and Wiesel were writing, see Wayman (2007, p. 266). A case study of the personal relationship to research objects in biology is provided by Fox-Keller (1983), see also the discussion in Knorr-Cetina (1997, pp. 16–20).

19 Nicholas Swindale (personal communication) pointed out that LeVay first chose the stripes of the mackerel as a comparison when he presented the anatomical reconstruction to the Royal Society circa 1975. If the above interpretation is plausible, the fingerprint provides a more powerful source for analogy.

20 It should be noted that the problematic status of this influential result is the main reason why Mountcastle’s strong anatomical modularity thesis was disputed later on (see Rakic 2008, for a defense, see Carlo and Stevens 2013). Curiously, numbers for visual cortex are doubled, which was neither explained by the Rockel et al. nor by Mountcastle. Also, neuron number is not the only quantitative fact that defines differences between cortical areas—the density and size of cortical layers can also be used to differentiate different parts of the cortex.

21 It is important to remember, however, that Mountcastle’s top-down (re-)organization of the columnar domain was retrospective in character. It was actually preceded by a lateral spread of the vertical electrode recording techniques to other research groups and experimental contexts.
